# ‘The importance of small things and colleagues’. Enhancing the wellbeing of healthcare staff: Appreciative Inquiry in an integrated UK maternity service

**DOI:** 10.1017/S1463423626101479

**Published:** 2026-08-28

**Authors:** Rachel Arnold, Edwin van Teijlingen, Preeti Mahato

**Affiliations:** 1 Centre for Midwifery and Women’s Health, Bournemouth University, Bournemouth, UK; 2 Royal Holloway Law School, Royal Holloway University of London, London, UK

**Keywords:** Appreciative Inquiry, collegiality and kindness, maternity workforce, psychological safety, recruitment and retention, staff wellbeing

## Abstract

**Aim:**

The aim of this study was to identify factors that enhanced staff wellbeing in an integrated UK maternity service.

**Background:**

Staff wellbeing affects the experience of patients, quality and safety of services, and staff retention. Global healthcare staff shortages mean we need to enable staff to thrive and remain in professional practice. High levels of stress, depression, burnout, attrition and staff shortages have been reported in maternity services. It is, therefore, critical to find ways to support the wellbeing of existing staff.

**Methods:**

Appreciative Inquiry interviews (n = 39) and group discussions (n = 4) were conducted with a cross-section of midwifery, medical and maternity support staff, working in community teams, the inpatient and midwife-led unit. Interviews explored best experiences, values and what made a difference to wellbeing. Data was co-analysed thematically by the research team, participants and midwifery staff.

**Findings:**

Despite workload and staffing pressures midwives, doctors, students and maternity support workers were highly motivated and passionate about their work. Five staff wellbeing themes were identified: Everything we need to do our work practically and psychologically; Meaning – in our work and relationships; Opportunities to learn and grow; Finding our niche; Agency – able to take care of ourselves. This study also revealed the important contribution that kindness, collegiality, feedback and psychological safety, made to staff wellbeing including professional confidence. Our findings suggest that every healthcare provider can make a difference to their colleagues wellbeing, contribute to the safety of the service and staff retention. An outline of practical recommendations for improving staff wellbeing is provided.

## Introduction

The wellbeing of healthcare providers affects patient experience, the quality and safety of services as well as the individuals (Hall *et al*., 2016; Hodkinson *et al*., 2022; Maben *et al*., 2012). Staff who are thriving are less likely to leave or retire early (Moncrieff *et al*., 2023). This is critical given the global shortage of health workers and challenges in recruitment and retention (Chamanga *et al*., 2025; World Health Organization 2023), prioritizing staff wellbeing also makes financial sense (Boorman, 2009). Many measures to support healthcare providers’ wellbeing have been developed, such as the UK NHS Health and Wellbeing Framework (NHS England and NHS Improvement 2021). Globally, however, COVID-19 put pressure on health staff who were already struggling with burnout and poor mental health (De Kock *et al*., 2021; World Health Organization 2022).

Midwives, obstetricians and students have reported high levels of dissatisfaction, stress, depression, anxiety and burnout (Bourne *et al*., 2019; Harvie *et al*., 2019; Oates *et al*., 2020). The UK is short of 2500 midwives, while poor retention and early retirement threaten to deplete the workforce further and increase the pressure on remaining staff (Care Quality Commission, 2023; Hunter *et al*., 2019; Royal College of Midwives, 2024)^,^. As midwifery services continue to face unprecedented challenges with the increasing complexity of maternity service users (commonly referred to as women), and the intense scrutiny of performance, it is vital to understand how to enhance staff’s wellbeing.

Wellbeing is a difficult to define, complex and contested concept (Dodge *et al*., 2012) and includes objective and subjective measures. It is more than physical and mental health, as it includes how a person feels about their life with the focus on happiness (hedonistic tradition) and on living life in a full and satisfying way (eudaimonic tradition) (Deci and Ryan, 2008). A recent review (Taylor *et al*., 2022, p: 60.) described health staff’s wellbeing as *a pressing and complex problem, influenced by many factors at individual, organisational, interprofessional and broader societal level’.*

This study was conducted during the COVID-19 pandemic (Sept 2020–May 2022). The aim was to understand what helped staff wellbeing, what supported them to stay well and working, and their ideas for improving wellbeing. Appreciative Inquiry (Bushe, 2011; Whitney and Trosten-Bloom, 2010) focuses on meaning, on the best, and what ‘gives life’. This gave staff the opportunity to focus on their successes, strengths, and their teams – providing a reprieve from the intense external and internal scrutiny of maternity services while exploring key factors in wellbeing. It is congruent with a study that has at its heart the desire to enhance staff wellbeing.

## Methods

The study site was an integrated maternity service in Southern England that included a continuity team, home birth team and an alongside midwifery-led unit with most staff working between community teams and hospital. The principal investigator, an ex-midwife who had not recently worked in the NHS, spent several weeks orientating in all service areas. The study was then designed in collaboration with senior midwives and staff. Ethical approval was obtained from Bournemouth University Ethics Committee and the NHS HRA. The Director of Midwifery and Neonatal Services and Clinical Director supported the research.

The research was advertised by word of mouth, at staff meetings, in their newsletter, and notices in staff areas. Information sheets were distributed to interested staff. COVID-19 guidelines regarding masks and distancing were carefully followed.

Appreciative Inquiry interviews (Reed, 2011; Whitney and Trosten-Bloom, 2010) were conducted with 39 staff, plus four focus groups with a total of 20 staff. The group discussions and most interviews (n = 33) were conducted in person if the unit co-ordinator considered that staffing was sufficient. Several interviews were interrupted and resumed later due to work demands. A broad range of self-selected staff consented – newly qualified and experienced midwives (n = 27), trainee doctors (n = 11), a consultant obstetrician, student midwives (n = 6) and maternity support workers (MSW n = 14). Many were in specialist teams, e.g. core night staff, continuity team, or home birth team. The first author (RA) conducted the group discussions and majority of interviews.

Staff were asked about their best experiences, the conditions that made these possible, and what they valued about their work. Participants were probed to detail their valued experiences, reflect on their achievements, what helped their wellbeing, and their aspirations – both for themselves and the service. Interviews were digitally recorded and lasted between 30 and 110 minutes.

Thematic analysis (Terry *et al*., 2017) was used. Each interview was transcribed, coded, integrated into categories and initial themes. RA analysed the majority of data – the rest of the team analysed some interviews and helped to refine themes. Initial themes were then shared and refined further with maternity staff so that they could agree, refute, add perspectives or ideas; in keeping with Appreciative Inquiry philosophy (Reed, 2011; Arnold *et al*., 2022).

## Findings

**Figure 1.**
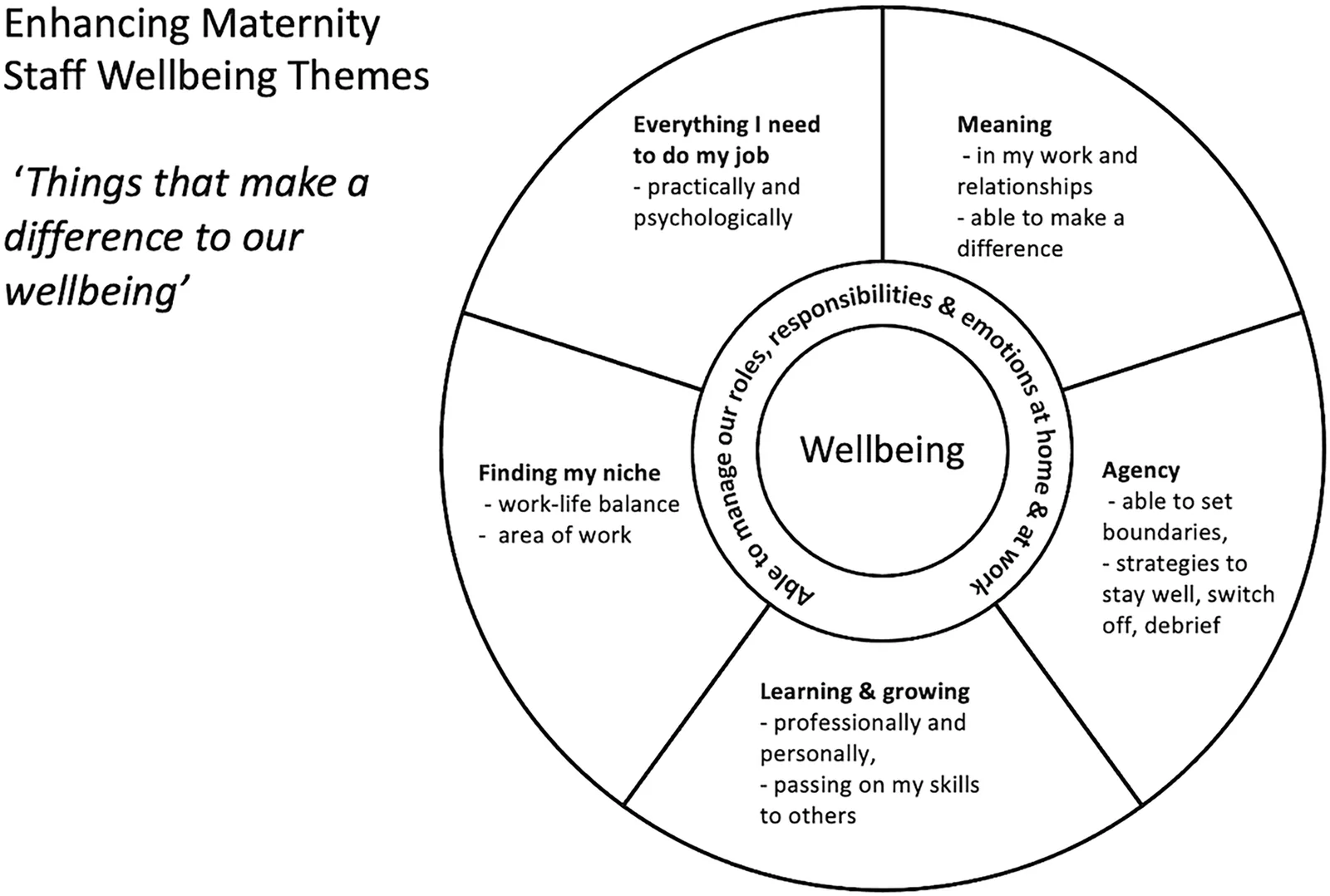
Enhancing maternity staff wellbeing themes.

Five overlapping themes (Figure 1) were identified: 1. Everything we need to do our job; 2. Meaning – in our work and relationships; 3. Opportunities to learn and grow; 4. Finding one’s niche; and 5. Agency – able to take care of ourselves. The overarching theme was ‘Managing roles, responsibilities and emotions at work and home’ (Arnold *et al*., 2024). This article focuses on the five wellbeing themes and their implications for staff’s wellbeing.

Despite workload pressures, staffing challenges, and COVID-19, staff were able to reflect on the satisfying and meaningful aspects of their work. This can be attributed to many factors: first local COVID-19 infection rates and resulting pressures were less than in other UK settings, secondly, in a small maternity service relationships are generally easier to establish, managers were also visible, approachable, flexible, acknowledging problems, even if they were unable to fix them. Many remarked on the difference that this made to morale.

### Everything we need to do our job

#### Practical needs met

Almost everyone said that ‘having enough staff’ was key for improving staff wellbeing. It affected everything, as more time could be spent with women in need, staff could take their breaks, stay hydrated and nourished, and take time off without feeling bad knowing the unit was short-staffed. In addition, they would not be called every time they were on-call, depriving them of sleep and their families. Staff explained how stressful it was trying to manage when shifts were short staffed and feeling apprehensive coming into work when a shift was understaffed.

Although ‘wellbeing’ and ‘adequate staffing’ were almost synonymous for staff, they acknowledged that it was ‘not just numbers’ that were important – it was also ‘*who you were on with*’. As a doctor explained:

The job is entirely made or broken for me by the people that I work with. IDI34

The implication being that some colleagues were more helpful and collegial than others. This was echoed by most – from student midwives to experienced doctors. There were many other practical issues that affected wellbeing such as getting breaks.

Breaks make a massive difference – it really helps to have eaten properly and had a bit of a break… these are really obvious simple things, but if you haven’t got them, it can be quite detrimental… Midwife IDI14

Several staff wanted a more restful place for breaks, somewhere conducive to switching off without work conversations, and not ‘decorated’ with work notices about things that could go wrong.

Practical needs involved having a uniform, and an ID badge on day one. It also included support returning after pregnancy or long-term sickness which several found stressful. They suggested re-orientation would be helpful – someone going through protocols or policy changes and extra support initially.

#### Psychological needs met

Midwives, doctors, students and MSWs needed to know that they were doing OK, that their contribution was valued, for example:

Some days you would kiss someone’s feet if they said, ‘I’ve seen how hard you’ve been working today’, … any kind of feedback… if they asked ‘are you OK…want a cup of tea?’ Newly qualified midwife IDI22

These words reveal an almost desperate need for validation, kindness and feedback. This was not confined to new midwives but was echoed by experienced midwives and doctors.

The good days are probably the days where someone says ‘Thank you’ …or [a senior colleague says] ‘well done you handled that well’… very simple things! Trainee doctors DG1.

Feedback also contributed to professional confidence:

I’ve always doubted myself, wondered what people thought of me and if I was good enough… I think my practice is fine but it’s difficult to… see that in yourself, so that’s why I think a bit of positive feedback goes a long way and could give people a morale boost that sometimes we need in this job. Experienced midwife IDI16

Staff emphasized how important it was to ‘feel safe’ to ask questions to more senior midwives without being made to feel stupid. Some days they felt less confident and needed to check - this was easier with some colleagues than others.

It’s an emotional thing … some people can make… you feel equal, respect[ed] – other people can make you feel belittled… it makes you feel… you don’t put it [show it] on the outside, but inside you think… Midwife IDI21

Feeling valued, and part of the team was vital for staff wellbeing. ‘*It’s a good feeling when we feel needed and part of the team’,* one MSW explained. Being included in conversations, listened to and acknowledged gave staff a sense of belonging and security. It reassured them that colleagues had their back. It helped if colleagues had a positive attitude regardless of the workload and, in addition, staff said they could not overstate the importance of having someone to off load with after a tough shift.

#### Management

The attitude and approach of management affected staff wellbeing, such as acknowledging problems – even if nothing could be done about them.

Even if they can’t do anything, a doctor explained, just to have someone say ‘yes I understand it is rubbish isn’t it?’ …it’s really huge to feel listened to. Doctor IDI34

The midwifery management team was visible and accessible. Their door was generally open, and staff of all grades said they could go with any concerns and be listened to.

I think it’s lovely… I’m only a first-year student, you feel like you’re bottom of the pack, but when you see management they always make you feel really welcome – like you’re really important. Student midwife DG4

Several trainee doctors were grateful for professional and personal support from midwifery managers – as well as the sense of team and being able to discuss complex clinical cases with the co-ordinators. The managers all worked clinically and if necessary, would ‘roll up their sleeves’ and help. This helped to solidify the sense of team.

…they [=management team] will put scrubs on and help whenever they need to… I think that massively boosts staff morale Midwife 2 DG4

When there were family crises, managers were flexible and tried to ‘create’ solutions so that staff could manage home and work.

They’re really quite accommodating… [management] know we have families – and families generally come first. They get that and it’s really, really important. Midwife 1 DG4

The managers supported ongoing training and tried to accommodate staff who were keen to work in specific areas. This contributed to a work environment where staff felt appreciated and valued. A midwife explained:

…here they look after their staff better [than other places she had worked], they worry about their staff wellbeing … when there’s not a lot of people to fill [posts], you need to retain your staff, and therefore you need to look after them. I think they do that here. Experienced Midwife IDI16

### Meaning

#### Meaning in our work

One thing that stood out was that staff of all grades wanted to know they were doing something meaningful – ‘making a difference’. This could be a ‘thank you’ from a colleague/parent, or being able to redesign part of the service to increase support to a vulnerable group – everyone needed to know that they were making a difference to someone.

…that smile on her face before she went home… you feel, you walk away and think I’ve actually helped someone today! Senior midwife IDI16

The need to make a difference was illustrated by the emotions that accompanied the stories. A student midwife on her first placement shared a special experience.

…the husband… didn’t speak much English, but he put his hand on my hand and said ‘thank you’ and he really meant it. And I was really emotional – [I knew I had] made a difference… Student midwife DG4

This was the first time she felt she had made a difference to someone – it was very moving for her. Similarly, one MSW explained:

So, anything where I feel I’ve made a difference – even a midwife saying ‘thank you so much, this has really helped’ … is a good day. MSW IDI33

Another source of meaning for staff was relationships.

#### Meaning in relationships

Midwives regarded the close connection with women and their families as one of the most satisfying parts of their work. As one of the home birth team explained:

I love the relationship with women and families. Delivering my own women – they know me, and I know them – it’s really magical, really lovely. Midwife IDI5

It was not only the home birth or continuity team, even for brief moments during antenatal consultations, support during childbirth or postnatally the sense of connection and trust was seen as a privilege that gave meaning and fulfilment. Another midwife explained, ’*The human connection is the motivator’*. While this was often with women – the connection with colleagues was also vital:

I love working here… the camaraderie [with] the women and the midwives and everybody that I hang out with, I’ve got some really good friends here. Student midwife IDI20

Some spoke of colleagues as family. *I feel this is my second home, my second family,* a senior doctor explained. Another said how much being valued and trusted meant:

I felt really valued… which I really enjoyed… when people get to know you, they know what you are capable of and there’s that trust between you – that when I say, ‘this is happening’, they know that I know what I’m talking about. … I really got a lot of out of that. Doctor IDI34

The other key relationships for maternity staff were their families. They talked about those at home who were proud of them and supported them. These ‘unseen’ relationships were vitally important in sustaining and giving meaning to these maternity staff lives.

### Opportunities to learn and grow

Learning something, acquiring and using new skills, or being sent on a course was very satisfying. ‘*We are here to learn and develop’,* one MSW explained. A registrar defined a good day as one where he had learned something - ‘*if someone explains something to me and it’s new for me, I feel excited and I’m happy’*. Many midwives talked about their love of learning, and growing in their skills and knowledge to be the best they could be – as several said *‘Every day is a school day’.*

Not only were they growing professionally, staff also enjoyed a sense of personal growth. Caring for women and working with colleagues with different life experiences and perspectives stretched them and helped them to grow. Part of learning was also the satisfaction of passing on knowledge as a midwife explained, ‘*seeing a student grow and change – that’s rewarding’.*

### Finding one’s niche

A key factor in staff thriving was having a congruity between work and their life experiences, interests, personalities and passions – as well as a good fit between working hours and family commitments. Some were proud to be competent in all areas of the integrated service, others had found their niche working in a specific team or speciality.

Now I’m in the role I really enjoy I… don’t have that many really bad days anymore. … this job allows me to do what I feel passionate about. Midwife IDI14

Several MSWs explained that ‘*We have chosen this role for a reason’.* Although they loved learning on the job, they were content and did not want to do more training. It fitted well with their busy home lives. Some midwives and doctors dreamt of specializing and were working towards it. One midwife had made the difficult decision to give up her community caseload and go on the bank but was amazed at the difference it made.

The difference it made to me is amazing! I’ve more energy, am less stressed and happier in my job. Midwife IDI8

A midwife who only worked a few shifts felt guilty for not doing more, but it was all she could manage. When at work, however, she could give 100% and she loved it.

There was no ‘one size fits all’ – but it was striking to see the energy and enthusiasm of staff who felt that their work arrangement was a good fit. Those staff were working the hours that suited them, in the speciality that fitted their skills and passions. One midwife who had recently found her niche, declared she was ‘*happy to stay until I’m old!*

### Agency

Many said how important it was to find ways to unwind and switch off at the end of a shift. Strategies varied but after a challenging shift having someone to off load with was an essential part of staying well. Many were on WhatsApp groups with peers or their team.

I’ve got two colleagues… I WhatsApp and can rant and they’ll rant back – that’s all I need. They’re on my wavelength – I don’t need to do details; I could just say ‘it’s been one of them!’ Midwife IDI17

Although going home to a loved one helped staff switch off, when they needed to talk through a tough day family were usually not so helpful as one doctor explained: *‘My husband tries and I love him to bits… but he doesn’t get it, he doesn’t understand’*!

Staff admitted they were *‘rubbish’* at debriefing themselves – being able to talk to colleagues who ‘got it’ was, therefore, invaluable. Two trainee doctors recalled a challenging delivery where they needed to call the consultant. Afterwards the consultant helped them reflect on the incident and did some teaching. ‘*It was brilliant’* one of the doctors explained, ‘*I actually went home fine’.*

Such support was vital for students and newly qualified midwives:

Sometimes I think I can’t do it, but peer support makes the difference. I lean on them a lot. Newly qualified midwife IDI19

Occasionally staff needed to talk to a more experienced colleague, and some suggested the need for a buddy system or counselling.

Being self-aware and able to take care of themselves was also part of staying well several explained.

I think we have to take responsibility for ourselves and say ‘yes – I’m going to take a break’ because working on a very low sugar and very low energy is…[tough]. Midwife IDI14

A trainee doctor said it’s ‘*almost a badge of honour*’ – not eating all day, normalising the stress, the isolation, and feeling they are expected to put ‘patients’ before their own needs. One of the midwives said her team was very supportive – but then admitted she didn’t always ask when she needed help. ‘*It’s about asking… sometimes we have days where we struggle, and we don’t always ask’.*

Table 1 shows staff recommendations for enhancing their wellbeing grouped by theme and the contribution of various stakeholders.

**Table 1.** Maternity staff recommendations for enhancing their wellbeing Table 1 long description.

	Everything we need to do our job practically & psychologically. We need:	Meaning – in our work & relationships, knowing we have made a difference	Agency – able to set boundaries & say ‘no’, strategies to switch off, someone to debrief with.	Learning & growing professionally & personally, teaching others	Finding our niche – area of work and work–life balance
**Government, NHS, Trust**	Enough staff with right skills/experience. (This will keep the service safe & reduce workload, pressure & stress we feel when we are short staffed). Well-funded visible & easily accessible wellbeing/staff support services	Enough staff will help us give women time they need – and know we are making a difference every day. Revise NHS wellbeing framework to link working environment to wellbeing.	Enough staff will mean we can take our breaks, reduce felt pressure to work extra shifts, stay late, work in our holidays. We will then have a more balanced life, recharge emotionally & physically, & have more to give when we work.	Enough staff will enable us to attend training days more regularly & have the time & energy to do updates & pass on my skills to others.	Enough staff will reduce the pressure we feel to work extra shifts, stay late, work our holidays. We will then have a more balanced life, recharge emotionally & physically & have more to give when we work.
**Managers & senior colleagues, Professional Midwifery Advocates (PMAs)**	Managers who are visible, available, listening, acknowledge problems even if they can’t fix them, & flexible when I have a crisis at home. Senior colleagues/PMA’s we can easily access for advice/support/debriefing	Being acknowledged & thanked/ knowing I have made a difference is really important for us.	We need more experienced staff/PMAs for professional support, or who offer debriefing after a challenging incident.	We need managers who offer us opportunities to do extra training Feedback from managers helps build our professional confidence	Managers who help us work in the areas we feel the most passionate about/skilled in. Managers who try to adjust our working hours when necessary.
**Co-ordinators, and experienced colleagues**	Shift co-ordinators & colleagues who are friendly & approachable, who welcome ideas & don’t make us feel stupid for asking basic questions	Colleagues, (service users), managers who thank us and help us know we have made a difference.	More experienced staff we can go to for debriefing, or who offer debriefing after a challenging incident.	More experienced staff who help us develop new skills, share insights/discuss current research with us.	Managers & senior staff who recognize what we are good at and help us see it also.
	Feedback from shift co-ordinators and colleagues to help build our professional confidence			Feedback from shift co-ordinators & colleagues helps us learn, & builds our professional confidence	
	Positive, friendly atmosphere regardless of workload				
**Peers/colleagues midwives, trainee doctors, MSW’s, student midwives**	Feedback from colleagues to reassure us & build our professional confidence	Colleagues (& service users) who thank us & help us know we have made a difference.	Peers & colleagues we can debrief with (informally) after a tough shift	Colleagues, peers who teach new skills, share insights/ discuss current research	Colleagues who recognize what we are good at & help us to see it also.
	Positive atmosphere, humour & support				
	Teams where we feel safe, valued, included. A team that looks out for each other – especially during tough times.	Colleagues who notice when I’m having a tough day – who ask, offers a drink/break.	Peers & colleagues we can do fun things with outside of work		
	Teams that welcome our thoughts and ideas – regardless of our role				
**Individual staff** **(self-care)**	I need to build a sense of team with colleagues – with my peers & colleagues from other professional groups. This will improve the quality of our work together, help my wellbeing & theirs.	I need to build a sense of team with colleagues – to look out for them, be willing to help, approachable & kind. This will improve the quality of our work, my wellbeing & theirs.	I need to learn to say ‘no’ sometimes, to ask when I need help, to take breaks, to find strategies that help me switch off, & recognize when I need help to debrief & find support.	I need the courage to ask questions no matter how basic, to ask for support when I need it, learn from tough incidents and go on learning.	I need to reflect on my work, & work life balance occasionally. To see what brings me joy, identify my biggest struggles & determine if I need to make adjustments.

Many wellbeing elements were common to all staff – the need for human kindness, inclusion, feedback and help to debrief after challenging shifts. Staff were often surprised when this commonality emerged in group discussions and ongoing conversations. The different stressors and assets of each profession and career stage, however, resulted in slightly varying areas of vulnerability and wellbeing needs as portrayed in Table 2.

## Discussion

The wellbeing themes identified align with many concepts in the NHS Health and Wellbeing Framework (NHS England and NHS Improvement, 2021) which acknowledges that wellbeing encompasses mental, emotional, physical wellbeing, and a healthy lifestyle. Our findings concur with the importance of fulfilment at work, of skilled managers with healthy leadership behaviours, supportive relationships with colleagues, professional wellbeing support, and the steps that individuals can take to improve their wellbeing. Similarly, the importance of leadership support and intrinsic motivators was also highlighted in a study exploring recruitment and retention of adult community nurses (Chamanga *et al.*, 2025). The Kings Fund ‘Courage of Compassion’ report (West *et al*., 2020) identified three core needs to ensure wellbeing at work and minimize stress – ‘Autonomy, Belonging and Contribution’, which largely overlap with our themes – apart from the importance of learning and growing which appears to be missing.

Our findings also align with the wider psychological wellbeing literature – the importance of a sense of purpose, satisfying relationships, autonomy, personal growth and self-realization (Ryff, 1989). The eudaimonia tradition of wellbeing suggests that having a sense of fulfilling one’s virtuous potential and living as one was inherently intended to live is important for wellbeing (Deci and Ryan, 2008). Making a difference was vital for the psychological wellbeing of all in our study, it was deeply satisfying, motivated them, and gave a sense of pride or as one midwife explained, the assurance that ‘*my life has meaning’* (Arnold *et al*., 2024). The staff were exemplars of the power of meaningful work to give courage, determination, and joy even in a pandemic. The struggles of MSWs who had lost some of their more fulfilling roles also illustrates the importance of meaningful work for wellbeing.

Humans need work that is significant, has a broader purpose and aligns with their values (Martela and Pessi, 2018). This could be seen in staff who struggled when they could not give care according to their standards and values. This causes distress and contributes to attrition (Cull *et al*., 2020; Harvie *et al.,* 2019). The NHS Health and Wellbeing Framework, however, misses the link to the working environment and personal values (Martela and Pessi, 2018). Staff in our study highlighted that wellbeing is inextricably linked to having everything they need to give high standard care such as enough appropriately skilled staff (Sandall *et al*., 2016).

### Individual staff

Although individuals can help their own wellbeing by setting boundaries, self-compassion (Beaumont *et al*., 2016), developing strategies to switch off, knowing their limits and asking for help – the majority of wellbeing factors relate to the working environment. Burnout, for example, has been recognized as an organizational rather than an individual issue (Montgomery *et al*., 2019). The primary responsibility and opportunity to improve staff wellbeing lies with government, NHS, management and colleagues.

### NHS maternity organization, & management

Any discussion of the wellbeing of maternity staff in 2026 must highlight the structural problem of staff shortages (Royal College of Midwives, 2024; Taylor *et al.,* 2022; World Health Organization, 2023). Enough staff has to be the bottom line for both staff wellbeing and safety of services (Moncrieff *et al.*, 2023). To suggest that any other wellbeing initiative can make up for the lack of staff does a disservice to those using every strategy, resource, skill and ounce of energy to provide high-quality, safe maternity care for every woman every day.

*The single most… powerful influence on the culture of… organisations is leadership (West et al., 2014, p: 241).* Poor relationships with managers affect wellbeing and contribute to intentions to leave the profession (Harvie *et al.,* 2019; Moncrieff *et al.*, 2023). Although the impact of management on wellbeing was outside the remit of this study many participants pointed to leaders who were both compassionate, and effective (de Zulueta, 2016). These managers acknowledged everyone, were present, approachable, proactive in addressing problems, invited feedback and worked clinically if needed. Staff said they felt valued, listened to, and supported. This helped their wellbeing and provided a cultural model for the service (Schein, 2010).

### Colleagues

Reports into failings in maternity services often include accounts of bullying, and toxic environments (Kirkup, 2015; Ockenden, 2022). Poor relationships with colleagues and management contribute to the staff attrition (Moncrieff *et al.,* 2023). Staff in our study ‘turned this on its head’ – by revealing the positive impact that colleagues made to their stress, anxiety, mental health and professional confidence. Knowing that colleagues had their back – reduced midwives’ stress because they felt part of a supportive team rather than working in isolation. Feeling connected, noticed, and supported is particularly important for student midwives and newly qualified midwives (Bacchus and Firth, 2017; Oates *et al.,* 2020). Feeling connected to the midwifery team was important for trainee doctors and reduced the sense of isolation that is detrimental to their mental health (Carrieri *et al*., 2020). Receiving feedback helped students, midwives and doctors grow in their professional confidence. This concurs with Bedwell *et al*. (2015) who found that midwives’ intrapartum care confidence was fragile and that colleagues were key in building confidence. Having peers to off load with and colleagues to talk through clinical incidents reduced stress and anxiety. This is vital for maternity services staff and is a role that colleagues are uniquely positioned to fulfil (Cull *et al*., 2020; Hunter *et al*., 2019). Cramer and Hunter (2019) highlighted the link between emotional wellbeing and the quality of relationships with colleagues. This concurs with our findings – not only for midwives but all maternity staff.

Insufficient staff (87%) was the top reason for midwives’ intention to leave the profession in the REMAIN study (Moncrieff et al., 2023). Reasons also included working culture and relationships: not feeling valued by management or colleagues; culture at work; and relationships with colleagues. Our study shows the potential that all staff have, to enhance their colleagues’ wellbeing and improve staff retention.

Feeling safe to ask questions without the fear of being belittled reduced staff members’ stress. This ‘psychological safety’ – ‘*the belief that the workplace is safe for speaking up – with ideas, questions, concerns and even mistakes’* lays the foundation for effective learning and contributes to a safer organization (Edmondson, 2003; The King’s Fund, 2020) Furthermore, trainee doctors brainstorming with co-ordinators strengthened the sense of team and interdependency across professions. This contributes to ‘collective competence’ an important mechanism for safety that includes ‘*strong social ties, interdependency… and professional boundaries that are managed flexibly with a deference to expertise rather than hierarchy’* (Liberati *et al*., 2021, p: 445). Positive relationships, therefore, make a difference to patient outcomes and the safety of services (Braithwaite *et al*., 2017)

Appreciative Inquiry enabled staff to acknowledge the difference that colleagues made to their wellbeing. Furthermore, they discovered that supportive relationships, positivity, humour, feedback and seemingly insignificant kindnesses were important to all. We contend that collegiality and supportive relationships exist to varying degrees in all maternity services – but that they are only visible, validated, celebrated and multiplied when strengths-based methodologies are used. Liberati *et al.* (2021) noted the importance of illuminating these relatively low-resource changes that might make a difference for patient safety, [that] might otherwise remain invisible or be regarded as ‘fluffy’ (Liberati *et al.*, 2021, p: 454). Furthermore, although there were unique wellbeing challenges for each profession, we saw the value of conducting a cross-disciplinary wellbeing study because it strengthened relationships and the sense of shared humanity regardless of roles.

Although there are unique challenges for healthcare worker wellbeing in other global settings (The Lancet Global Health, 2023) such as low- and middle-income countries, conflict areas, we suggest that interpersonal kindness, collegiality and managerial flexibility for this predominantly female workforce may well contribute to their wellbeing also.

## Strengths and limitations

This research was conducted in a small UK maternity service. It would be beneficial to replicate it in larger maternity services, primary health care services, other specialities and different cultural settings to ascertain the similarities or differences in wellbeing needs. The pressures of COVID-19 resulted in a study that was primarily done in working hours, was opportunistic, and sometimes interrupted by the needs of the service, making it difficult to always complete the full Appreciative Inquiry cycle. The conversations around meaning and things that ‘gave life’, however, were in keeping with its philosophy.

Appreciative Inquiry was a strength, creating a safe space for staff to reflect on the meaningful aspects of their work – things that brought life to them. It also highlighted, validated and strengthened what was obvious but unseen – the positive contribution that collegiality and kindness was making to staff wellbeing. This changed conversations and strengthened the good that was already happening. This was unlikely to have occurred with other methodologies.

## Conclusion

The things that made a difference to staff wellbeing were having: meaning in work; everything they needed to do the job – practically and emotionally; agency to set boundaries and employ strategies to stay well; opportunities to learn and grow; and finding their niche.

What is more unusual is that our findings highlight the potential of collegial relationships to enhance staff wellbeing, reduce stress, anxiety and isolation, build professional confidence and potentially improve staff retention. In addition, it suggests that by welcoming questions however basic, managers, team leaders and individual staff can help to create a psychologically safe environment leading to a safer maternity service.

The Appreciative Inquiry illuminated things that staff were already doing to enhance their colleagues’ wellbeing. Crucially it stimulated ‘new’ conversations between staff of all grades and roles that validated the importance of these seemingly small things, which consequently encouraged more of these behaviours and generated new initiatives. It helped staff realize that everyone can contribute to the wellbeing of their colleagues.

Our findings do not negate the need for structural improvements – especially funding, long-term planning and provision for more staff and the need for compassionate, effective leadership. They do, however, highlight that every team member has the power to make a difference to the working environment, the wellbeing of colleagues, and the safety of their maternity service.

**Table 2.** Variations in professional wellbeing needs Table 2 long description.

Doctors	Midwives	Student midwives	Maternity Support Workers
More isolated work & generally brief involvement with women, therefore, camaraderie with midwifery team & coordinator really valued	Keeping everyone safe, working to high standards & developing profession confidence requires being able to ask questions. A friendly supportive environment where everyone feels safe to ask questions, regardless of experience, was therefore, key. It also reduced midwives stress	Midwifery training intensely challenging, therefore, a friendly supportive environment where students feel safe to ask questions & feel part of team is vitally important	Some fulfilling aspects of their work had been lost, they therefore, wanted more opportunities to make a difference including more contact with women
Feedback rare therefore very meaningful – whether from service users, peers, midwifery team or consultants	Affected by workload and staffing pressures. Well-staffed shifts give midwives the satisfaction of working to their personal values & knowing they are making a difference		Being thanked & feeling part of the team of very important

## Data Availability

Research participants for this study did not consent for their data to be made available to other parties other than the research team.

## Table 1. Long description

The table has six rows and five columns. The columns are labeled 'Everything we need to do our job practically & psychologically. We need:', 'Meaning – in our work & relationships, knowing we have made a difference', 'Agency – able to set boundaries & say no’, strategies to switch off, someone to debrief with.', 'Learning & growing professionally & personally, teaching others', 'Finding our niche – area of work and work-life balance'. The rows are labeled 'Government, NHS, Trust', 'Managers & senior colleagues, Professional Midwifery Advocates (PMAs)', 'Co-ordinators, and experienced colleagues', 'Peers & colleagues midwives, trainee doctors, MSW’s, student midwives', 'Individual staff (self-care)'. Each cell contains specific recommendations and contributions from various stakeholders.

Navigate back to Table 1..

## Table 2. Long description

The table has four columns and four rows, comparing the professional wellbeing needs of doctors, midwives, student midwives, and maternity support workers. The columns are labeled Doctors, Midwives, Student midwives, and Maternity support workers. The rows describe various aspects of their work and needs. Row 1: Doctors: More isolated work and generally brief involvement with women therefore. Midwives: Workload pressures and staffing challenges. Student midwives: Midwifery training intensely challenging therefore. Maternity support workers: Some fulfilling aspects of work had been lost, therefore needed. Row 2: Doctors: Camaraderie with midwifery team and coordinator really valued. Thanks from women and colleagues particularly appreciated. Midwives: Well-staffed shifts give midwives the satisfaction of working to their personal values and knowing they have made a difference. Student midwives: Friendly supportive environment to feel safe to ask questions and feel part of team are vitally important. Maternity support workers: More opportunities to make a difference including more contact with women. Row 3: Doctors: Feedback rare therefore. Midwives: Keeping everyone safe and working to high standards and developing profession confidence requires being able to ask questions, therefore. Student midwives: Some aspects of their work less satisfying. Maternity support workers: Feedback very meaningful from peers, midwifery team and consultants. Row 4: Doctors: Friendly supportive environment where everyone feels safe to ask questions regardless of experience was key. It also reduced midwives stress. Midwives: Being thanked and feeling part of the team, therefore, of extra importance.

Navigate back to Table 2..
